# Treatment with Caffeic Acid and Resveratrol Alleviates Oxidative Stress Induced Neurotoxicity in Cell and *Drosophila* Models of Spinocerebellar Ataxia Type3

**DOI:** 10.1038/s41598-017-11839-0

**Published:** 2017-09-14

**Authors:** Yu-Ling Wu, Jui-Chih Chang, Wei-Yong Lin, Chien-Chun Li, Mingli Hsieh, Haw-Wen Chen, Tsu-Shing Wang, Chin-San Liu, Kai-Li Liu

**Affiliations:** 1 Department of Nutrition, Chung Shan Medical University, No. 110, Sec. 1, Chien-Kuo N. Rd., Taichung, 40203 Taiwan; 2 Vascular and Genomic Center, Changhua Christian Hospital, Changhua, 50094 Taiwan; 3 Graduate Institute of Integrated Medicine, College of Chinese Medicine, China Medical University, No.91, Hsueh-Shih Road, Taichung, 40402 Taiwan; 4 Department of Medical Research, China Medical University Hospital, Taichung, 40447 Taiwan; 5 Department of Nutrition, Chung Shan Medical University Hospital, Taichung, 40203 Taiwan; 6 Department of Life Science and Life Science Research Center, Tunghai University, Taichung, 40704 Taiwan; 7 Department of Nutrition, China Medical University, Taichung, 40402 Taiwan; 8 Department of Biomedical Sciences, Chung Shan Medical University, Taichung, 40203 Taiwan; 9 Department of Neurology and Vascular and Genomic Center, Changhua Christian Hospital, Changhua, 50094 Taiwan

## Abstract

Spinocerebellar ataxia type 3 (SCA3) is caused by the expansion of a polyglutamine (polyQ) repeat in the protein ataxin-3 which is involved in susceptibility to mild oxidative stress induced neuronal death. Here we show that caffeic acid (CA) and resveratrol (Res) decreased reactive oxygen species (ROS), mutant ataxin-3 and apoptosis and increased autophagy in the pro-oxidant *tert*-butyl hydroperoxide (tBH)-treated SK-N-SH-MJD78 cells containing mutant ataxin-3. Furthermore, CA and Res improved survival and locomotor activity and decreased mutant ataxin-3 and ROS levels in tBH-treated SCA3 *Drosophila*. CA and Res also altered p53 and nuclear factor-κB (NF-κB) activation and expression in tBH-treated cell and fly models of SCA3, respectively. Blockade of NF-κB activation annulled the protective effects of CA and Res on apoptosis, ROS, and p53 activation in tBH-treated SK-N-SH-MJD78 cells, which suggests the importance of restoring NF-κB activity by CA and Res. Our findings suggest that CA and Res may be useful in the management of oxidative stress induced neuronal apoptosis in SCA3.

## Introduction

Spinocerebellar ataxia type 3 (SCA3) or Machado–Joseph disease (MJD), a late-onset and fatal neurodegenerative disorder, is the most prevalent inherited ataxia worldwide. SCA3 shares features with other polyglutamine (polyQ) diseases such as Huntington’s disease and SCA7, which are caused by the expansion of a polyQ stretch in a disease-specific protein prone to aggregation and neurotoxicity^1^. In SCA3, the abnormal expansion of cytosine-adenine-guanine (CAG) repeats in the C-terminal coding region of the *ATXN3*/*MJD1* gene results in the expression of 55 to 87 glutamines in the mutant ataxin-3 protein. By contrast, a normal ataxin-3 protein contains 10 to 51 glutamine repeats. Ataxin-3, an intracellular protein of unknown physiological function, is widely expressed in the central nervous system and peripheral tissues. Expression of the mutant ataxin-3 protein in patients with SCA3 leads to selective neurotoxicity in restricted brain regions, which brings about a progressive loss of motor coordination, dysarthria, dysphagia, oculomotor dysfunction, and premature death^1^. Little is known about the specific targets of mutant ataxin-3. Although polyQ-expanded mutant protein aggregates are a hallmark of polyQ diseases, the involvement of ataxin-3 aggregates in the neurodegeneration of SCA3 is controversial^2^.

Oxidative stress, which is associated with increased reactive oxygen species (ROS) accumulation, is important in the pathogenesis of several late-onset neurodegenerative diseases. Oxidative stress results in injury to biological molecules and initiates neuronal apoptosis^3^. Mutant ataxin-3 is associated with decreases in antioxidant defence and a reduced capacity to deal with oxidative stress, which may play a crucial role in the neuronal cell death in SCA3^4^. Notably, compared with wild-type human SK-N-SH cells harboring normal ataxin-3, SK-N-SH-MJD78 cells containing mutant ataxin-3 are susceptible to declining GSH and total glutathione levels and reduced viability in response to *tert*-butyl hydroperoxide (tBH) treatment, which is commonly used to assess the involvement of oxidative stress in the pathogenesis of diseases^5–7^.

p53, a tumor suppressor and transcription factor, regulates many cellular processes, especially in apoptotic cell death^8^. There is a growing consensus that upregulation of p53 transcriptional activity plays a causative role in the mitochondrial-mediated neuronal apoptosis induced by the polyQ-expanded mutant proteins in SCA3, SCA7, and Huntington’s disease^9–12^. Ataxin-3 protein can de-ubiquitinate and stabilize p53. For example, injection of ataxin-3 mRNA leads to cell apoptosis in central neurons of zebrafish^13^. Notably, compared with normal ataxin-3 protein, the gain-of-function mutant ataxin-3 triggers higher p53 protein and p53-responsive gene expression and causes more severe p53-dependent neurodegeneration in brain neuron cells of transgenic zebrafish and SCA3 mice^13^. Moreover, mutant ataxin-3 increases the phosphorylation of p53 and enhances the transcriptional activity of p53, which in turn amplifies pro-apoptotic protein Bax expression in cultured cerebellar and pontine nuclei neurons^9, 10^. p53 inhibitor, pifithrin-α, prevents neuronal death in SCA3 transgenic mice and reduces mitochondrial membrane depolarization and cytotoxicity in PC12 cells expressing mutant huntingtin protein^9, 11^. Consequently, reducing the stability or activation of p53 may be a promising therapeutic strategy for SCA3 and other polyQ diseases.

Nuclear factor-κB (NF-κB) is a widely expressed transcription factor that is involved in several different cellular processes, including survival, apoptosis, proliferation, inflammation, and immune responses^14^. In the inactive state, the dimeric complex of NF-κB family proteins including RelA (p65), RelB, c-Rel, p50, and p52, resides in the cytoplasm by binding to inhibitory κB proteins (IκB)^14, 15^. NF-κB activation requires phosphorylation of IκB by the IκB kinase (IKK), which leads to degradation of IκB and then nuclear translocation and transcriptional induction of NF-κB target gene expression. Whether activation of NF-κB pathway in the central nervous system regulates neuronal cell survival or degeneration depends on experimental settings such as pathway stimuli, the type of cells, and the cellular environment^14, 15^. Notably, data have shown that phosphorylation of p65 facilitates transcriptional activity of NF-κB and is important in neuron growth, differentiation, and maintenance of synaptic plasticity^16^. Although it remains unknown whether the NF-κB pathway is involved in the neurodegeneration of SCA3, mutant huntingtin and ataxin-7 lessen the nuclear translocation and transcriptional activity of NF-κB, which results in a decrease in Bcl-x_L_ expression, caspase activation, and neuronal death^17, 18^.

Growing evidence from epidemiological and experimental studies has demonstrated the benefits of dietary polyphenols in brain health. Polyphenols exert these benefits through pleiotropic activities including antioxidant and anti-apoptotic effects^19^. Resveratrol (Res) is a polyphenolic stilbene that is widely distributed in red grapes, berries, and nuts^20^. Although Res has low bioavailability, it is able to cross the blood-brain barrier and has been shown to have neuroprotective properties in various neurodegenerative diseases, including polyQ diseases^20, 21^. Res improves motor deficits and imbalance in SCA3 transgenic mice and protects against cell death in primary cortical neurons containing truncated ataxin-3 with 79 glutamine repeats^22, 23^. However, whether Res has a protective effect on oxidative stress–induced neuronal cell death in SCA3 is unknown. Phenolic acids including hydroxybenzoic acids and hydroxycinnamic acids are the major representative member of polyphenols in plants. Caffeic acid (CA), classified as a hydroxycinnamic acid, accounts for almost 90% of total phenolic acid intake in the diet^24, 25^. CA is widely distributed in fruits, vegetables, wine, coffee, and olive oil and is readily absorbed from human small intestine and circulates in the plasma in millimolar concentrations^26, 27^. CA has been shown to have neuroprotective effects on cerebral injury, neurodegeneration, and neuroinflammation^28–31^. Notably, CA has a much more comprehensive profile of neuroprotection against various stressors than other members of the hydroxycinnamic acid family, such as chlorogenic acid, ferulic acid, and quinic acid^32^. Moreover, among the members of the hydroxycinnamic acid family, only CA can decrease hydrogen peroxide–induced oxidative stress and apoptotic cell death in cultured cerebellar granule neurons^32^. Given these antioxidant and neuroprotective properties of CA and Res, we investigated the effects and underlying mechanisms of CA and Res on tBH-induced neuronal cell death in both cell and *Drosophila* models of SCA3.

## Results

### CA and Res inhibit the effects of tBH on oxidative stress, cell viability, and cell apoptosis in SK-N-SH-MJD78 cells

**Figure 1:**
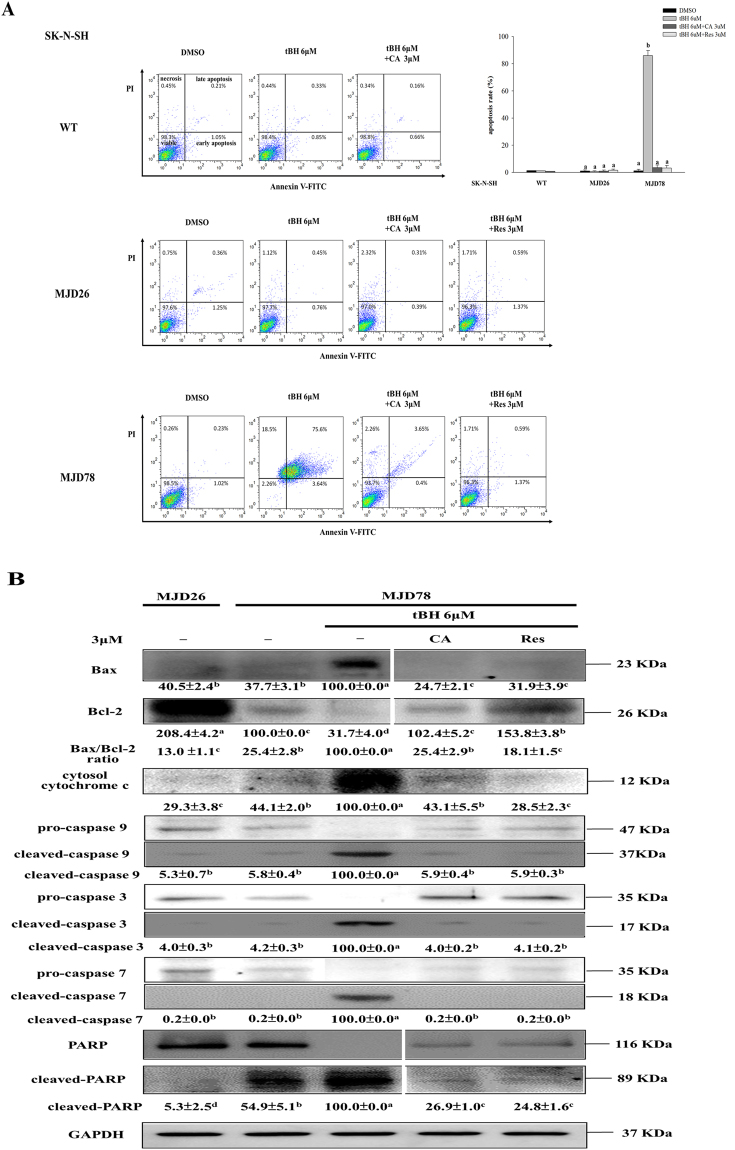
Effects of CA and Res on tBH-induced apoptosis in SK-N-SH WT, SK-N-SH-MJD26, and SK-N-SH-MJD78 cells. **(A)** Cell apoptosis was measured by flow cytometry (48-h treatment). Bar graph is presented as the percentage of early and late apoptosis defined as annexin V+/PI− and annexin V+/PI+. **(B)** Protein expression of Bax, Bcl-2, cytosolic cytochrome *c*, and pro and cleaved caspase 3, 7, 9, and PARP (24-h treatment). Data are mean ± SD and are expressed as the percentage of SK-N- SH-MJD78 cells treated with tBH alone. Values not sharing the same letter are significantly different (p < 0.05).

Previous data showed that SK-N-SH-MJD78 cells expressing mutant ataxin-3 with 78 glutamine residues are more sensitive to the effects of oxidative stress on cell viability than are parental SK-N-SH cells^5^. In agreement with previous results, we found that compared with treatment with the vehicle control, treatment with tBH significantly induced ROS production, cytotoxicity, and cell apoptosis in SK-N-SH-MJD78 cells but not in SK-N-SH cells or SK-N-SH-MJD26 cells expressing the normal ataxin-3 containing 26 glutamine residues. Moreover, compared with the vehicle control, tBH dramatically increased the events of mitochondria-mediated cell apoptosis as evidenced by significantly decreased mitochondrial transmembrane potential and Bcl-2 expression. These events occurred concomitantly with an increase in the expression of cytoplasmic cytochrome *c* and Bax as well as an increase in caspase 3 activity and cleaved caspase 9, caspase 7, caspase 3, and poly ADP-ribose polymerase (PARP) in SK-N-SH-MJD78 cells. Addition of CA and Res blocked the effects of tBH on ROS production, cytotoxicity, and cell apoptosis in SK-N-SH-MJD78 cells (Fig. 1 and Table 1).

**Table 1:** Effects of CA and Res on MTT assay, ROS, mitochondrial transmembrane potential and caspase 3 activity on tBH treated SK-N-SH WT, SK-N-SH-MJD26 and SK-N-SH-MJD78 cells^#^.

SK-N-SH	WT				MJD26				MJD78			
	DMSO	tBH 6 μM	tBH 6 μM + CA 3 μM	tBH 6 μM + Res 3 μM	DMSO	tBH 6 μM	tBH 6 μM + CA 3 μM	tBH 6 μM + Res 3 μM	DMSO	tBH 6 μM	tBH 6 μM + CA 3 μM	tBH 6 μM + Res 3 μM
MTT^&^	100.0 ± 0.0	94.5 ± 0.4	95.4 ± 0.3	95.5 ± 0.4	100.0 ± 0.0	99.8 ± 0.1	96.6 ± 0.3	99.1 ± 0.1	100.0 ± 0.0	43.4 ± 0.3	96.5 ± 0.2	94.7 ± 0.3
H_2_DCFDA^$^	4.5 ± 1.7^e^	7.9 ± 3.7^cde^			4.9 ± 1.4^de^	6.6 ± 3.3^de^			10.6 ± 0.3^bcd^	100.0 ± 0.0^a^	15.1 ± 3.4^b^	12.1 ± 2.3^bc^
MitoSOX^$^	0.8 ± 0.0^c^	0.7 ± 0.0^c^			0.8 ± 0.1^c^	0.8 ± 0.1^c^			4.3 ± 0.4^b^	100.0 ± 0.0^a^	4.1 ± 0.6^b^	4.4 ± 1.2^b^
TMRE^$^					4542.0 ± 13.5^a^				1552.0 ± 98.5^b^	100.0 ± 0.0^d^	1148.2 ± 3.0^c^	1150.1 ± 94.9^c^
caspase 3 activity^$^					8.6 ± 0.9^e^				32.1 ± 1.7^b^	100.0 ± 0.0^a^	28.7 ± 0.4^b^	45.6 ± 0.6^a^

^#^SK-N-SH WT, SK-N-SH-MJD26 and SK-N-SH-MJD78 cells were treated with or without tBH (6 µM) plus DMSO vehicle control, CA, Res (3 µM) for 48 h (MTT assay) or for 1 h (H2DCFDA) or for 3 h (MitoSOX or TMRE) or for 24 h (caspase-3 activity). Data are the mean ± SD of at least four separate experiments.

^$^Data are expressed as the percentage of the SK-N-SH-MJD78 cells treated with tBH alone and values in the same row with different superscript letters are significantly different (p < 0.05).

^&^Within same cell type, data are expressed as the percentage of cells treated with DMSO alone and values with different superscript letters are significantly different (p < 0.05).

### CA and Res weaken the effects of tBH on protein aggregate, mutant ataxin-3, and heat shock protein 27 (Hsp27) levels in SK-N-SH-MJD78 cells

**Figure 2:**
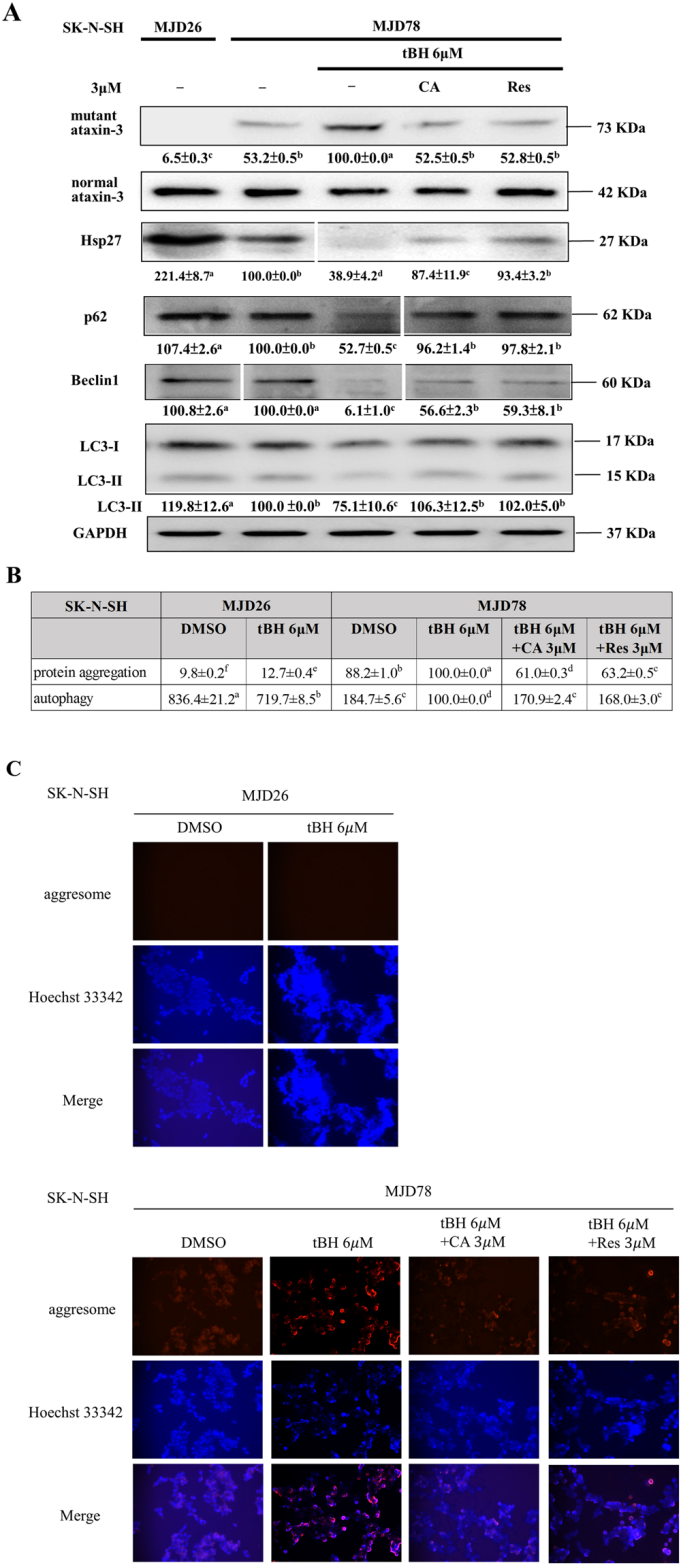
Effects of CA and Res on levels of mutant ataxin-3 and Hsp27, protein aggregates, and autophagy in tBH-treated SK-N-SH-MJD78 cells. After 24-treatment (**A**) Mutant and normal ataxin-3, Hsp27, p62, Beclin1, and LC3 protein expression were measured by Western blot analysis (**B**) Levels of Protein aggregates and autophagy stained by using aggregation assay, and acridine orange staining, respectively were quantified by flow cytometric analysis. (**C**) The images of protein aggregates were detected by ProteoStat Aggresome Detection Kit (Enzo Life Science (red). Cell nuclei were stained with Hoechst 33342 (blue). Data are the mean ± SD of at least four separate experiments and are expressed as the percentage of SK-N-SH-MJD78 cells treated with tBH alone. Values not sharing the same letter are significantly different (p < 0.05).

It has been established that the sensitization of apoptosis in neuronal cells with mutant ataxin-3 is associated with increased protein aggregates and decreased Hsp27 expression^33–35^. As shown in Fig. 2, compared with SK-N-SH-MJD26 cells, SK-N-SH-MJD78 cells showed increases in mutant ataxin-3 and protein aggregate levels as well as a decrease in Hsp27 expression, and these changes were augmented by tBH treatment. Addition of CA and Res significantly lessened the effects of tBH on the levels of protein aggregates, mutant ataxin-3, and Hsp27 in SK-N-SH-MJD78 cells (Fig. 2).

### CA and Res diminish the effect of tBH on autophagy in SK-N-SH-MJD78 cells

An increase in autophagy can reduce the expression of mutant ataxin-3 and protein aggregates in neurons, which results in modulation of neurodegeneration in SCA3 mice^35, 36^. In SK-N-SH-MJD78 cells, tBH treatment resulted in a decline in autophagy levels as measured by p62, beclin 1, and microtubule-associated protein 1 light chain 3 (LC3)-II protein expression and lysosomotropic agent acridine orange staining. Addition of CA and Res reversed the inhibitory effects of tBH on autophagy levels (Fig. 2).

### CA and Res impede the effect of tBH on p53 and NF-κB activation in SK-N-SH-MJD78 cells

**Figure 3:**
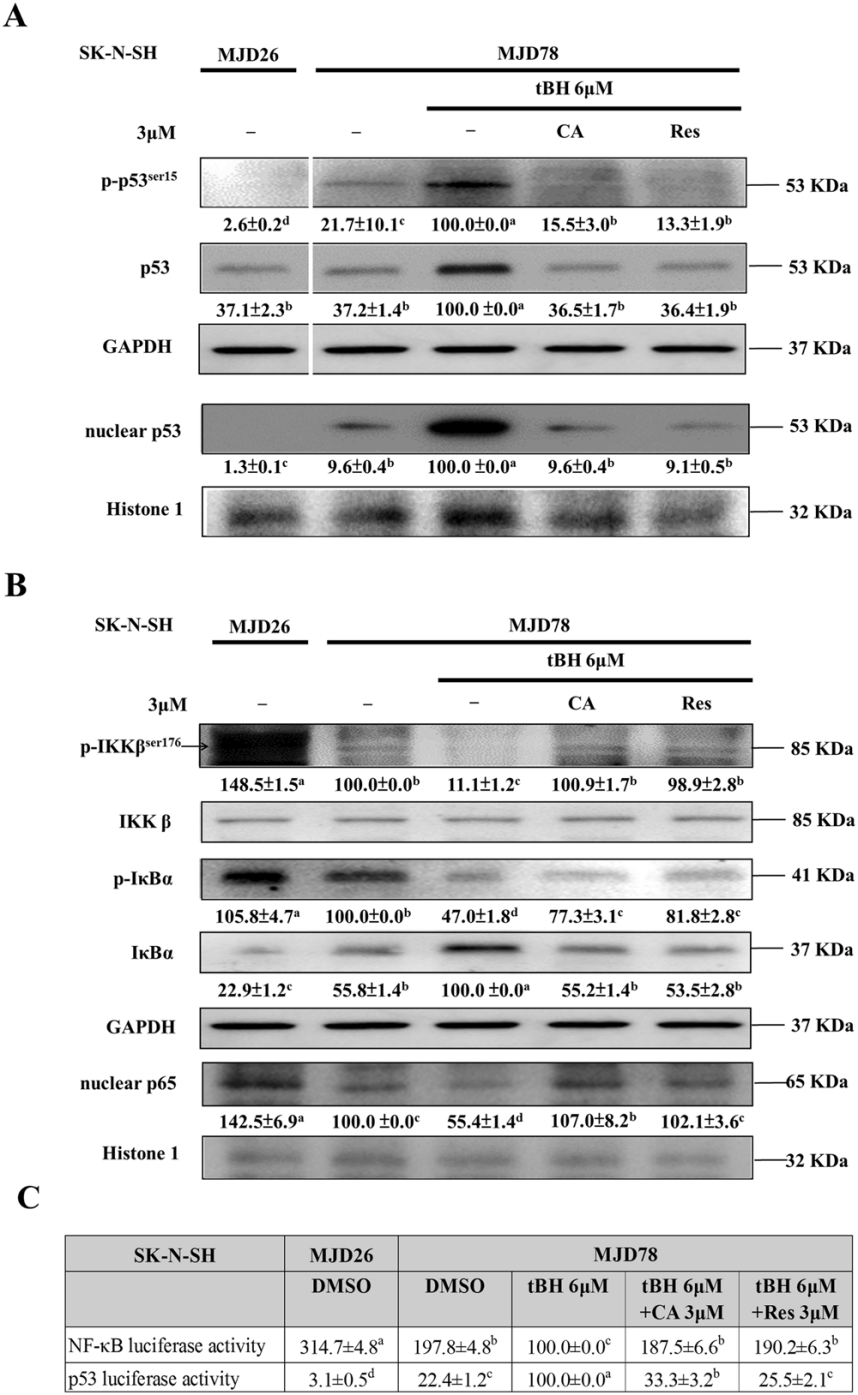
Effects of CA and Res on activation of p53 and NF-κB in tBH-treated SK-N-SH-MJD78 cells. **(A)** and **(B)** Protein expression of phosphorylated and total p53, IKK-β, IκB-α, and nuclear p53 and p65. **(C)** NF-κB and p53 reporter gene activities (3-h and 4-h treatments for p53 and NF-κB activation, respectively). Data are the mean ± SD and are expressed as the percentage of SK-N-SH-MJD78 cells treated with tBH alone. Values not having the same letter are significantly different (p < 0.05).

Treatment with tBH significantly induced p53 activation as evidenced by increases in total and phosphorylated p53 expression as well as nuclear p53 expression and p53 transcriptional activity in SK-N-SH-MJD78 cells (Fig. 3A and C). On the other hand, tBH significantly inhibited NF-κB activation through decreases in IKK-β and IκB-α phosphorylation, IκB-α degradation, nuclear p65 expression, and NF-κB transcriptional activity (Fig. 3B and C). Treatment with CA and Res abolished the effects of tBH on p53 and NF-κB activation in SK-N-SH-MJD78 cells (Fig. 3).

### Modulating NF-κB activity plays a critical role in the beneficial effects of CA and Res on tBH-treated SK-N-SH-MJD78 cells

**Figure 4:**
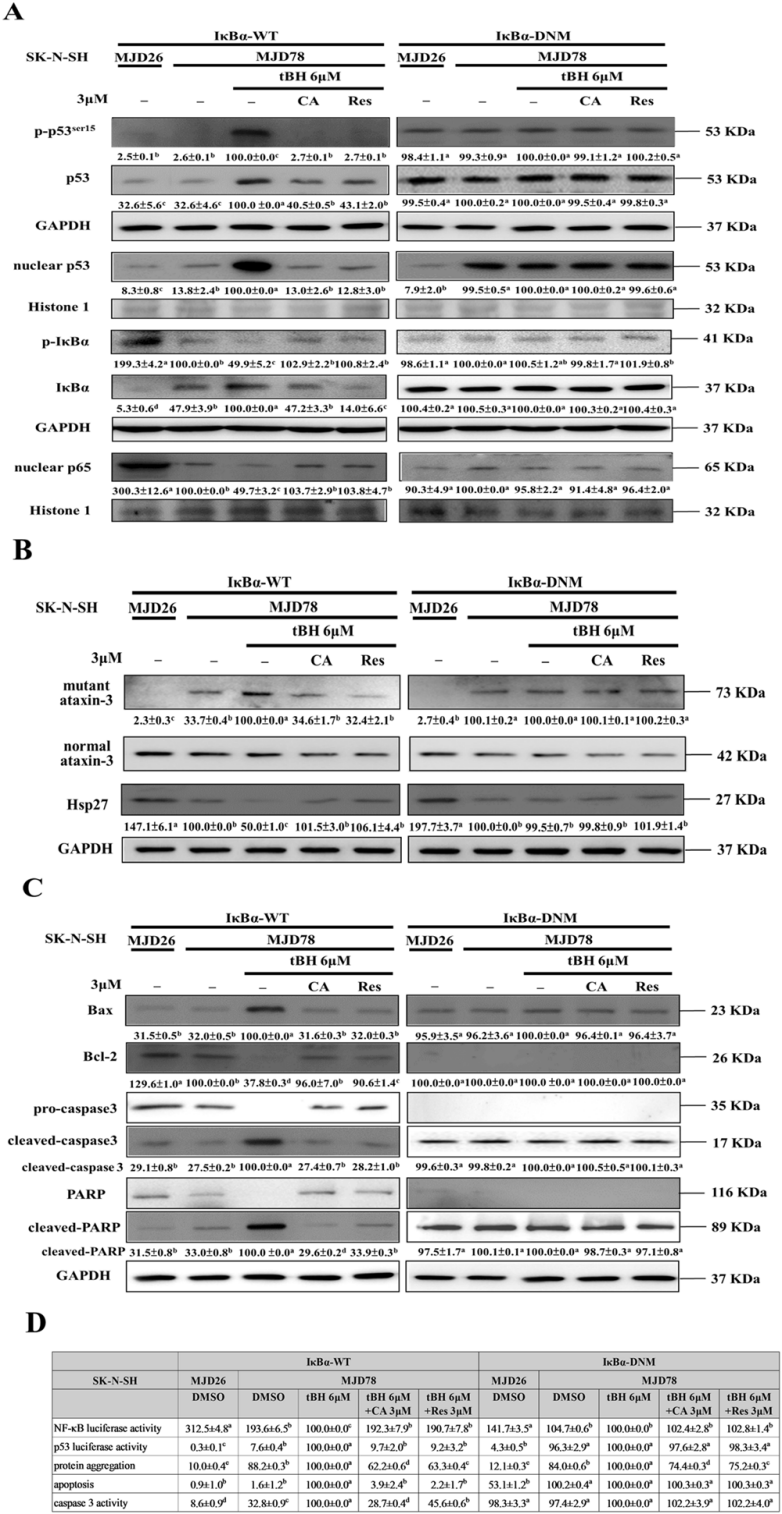
Effects of CA and Res in tBH-treated SK-N-SH-MJD78 cells transfected with dominant-negative mutant IκB-α. Cells were transiently transfected with IκB-α wild-type (WT) or DNM IκB-α as well as with or without reporter genes of p53-Luc or pNF-κB-Luc for 16 h and were then treated with either vehicle control or tBH plus CA or Res. **(A)** Phosphorylated and total p53 and IκB-α as well as nuclear p53 and p65 (3-h and 4-h treatments for p53 and NF-κB activation, respectively). **(B)** Protein expression of mutant and normal ataxin-3 and Hsp27. **(C)** Protein expression of Bax, Bcl-2, and pro and cleaved caspase 3 and PARP. **(D)** Levels of NF-κB and p53 reporter gene activities, protein aggregates, cell apoptosis rates, and caspase 3 activity. Data are the mean ± SD. Within treatments with the same plasmid transfection, data are expressed as the percentage of the SK-N-SH-MJD78 cells treated with tBH alone, and values not having the same letter are significantly different (p < 0.05).

We further performed transfection experiments with a dominant-negative mutant IκB-α (DNM IκB-α) that is resistant to phosphorylation and degradation of IκB-α and therefore acts as a potent repressor of NF-κB activation^37^. Transfection of DNM IκB-α into SK-N-SH-MJD78 cells blocked the ability of CA and Res to reverse the inhibitory effect of tBH on NF-κB activation (Fig. 4A and D). Moreover, the effects of CA and Res on the levels of protein aggregates, mutant ataxin-3, and Hsp27 with tBH treatment were negated when SK-N-SH-MJD78 cells were transfected with DNM IκB-α plasmid compared with the wild-type counterpart (Fig. 4B and D). Furthermore, the protective potency of CA and Res on tBH-induced cell apoptosis, apoptosis-related protein expression, and p53 activation was cancelled in SK-N-SH-MJD78 cells transfected with DNM IκB-α (Fig. 4A,C and D).

### CA and Res improve survival rates and climbing activity in tBH-treated ELAV-SCA3tr-Q78 transgenic *Drosophila*

**Figure 5:**
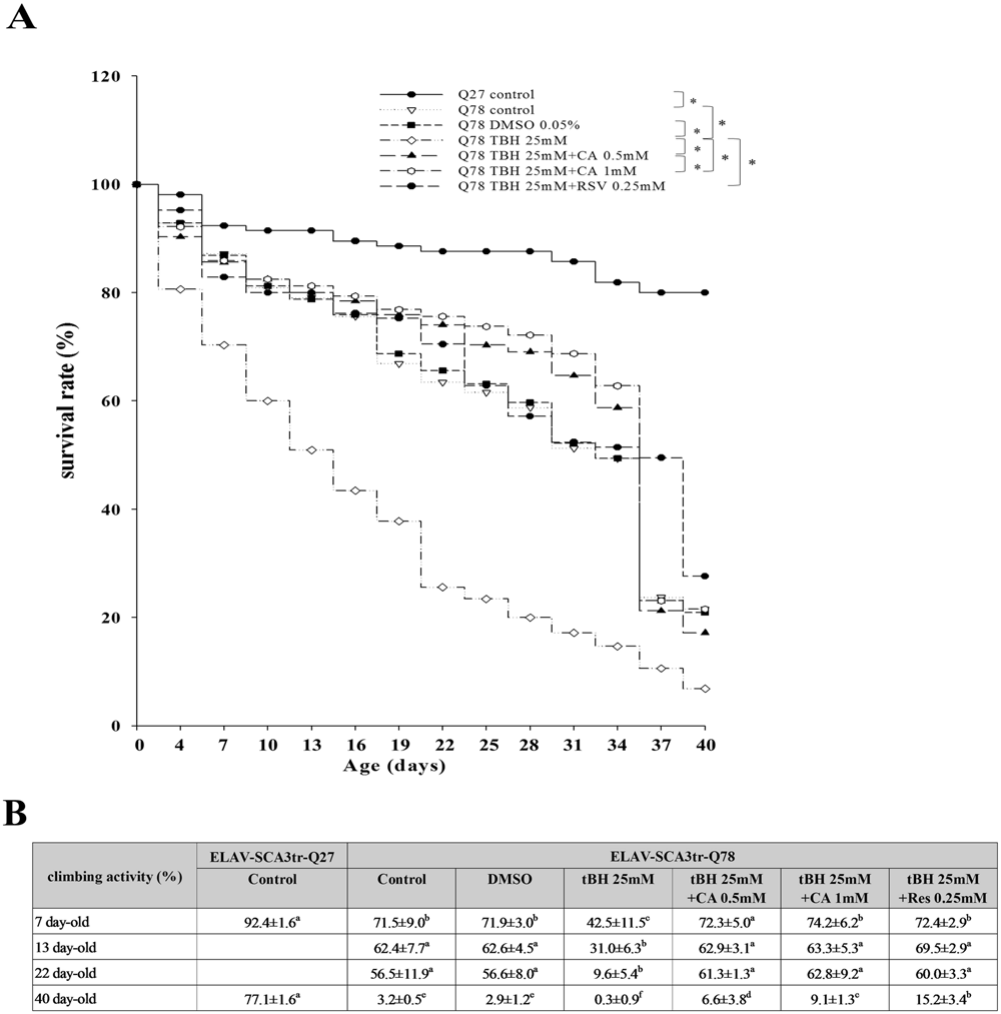
Effects of CA and Res on survival rates and climbing activity in tBH-treated ELAV-SCA3tr-Q78 transgenic *Drosophila*. **(A)** Survival rates were plotted and compared across groups by use of Kaplan-Meier log-rank analysis. The mean life span and SD are shown, *p < 0.01 (n = 300). **(B)** Climbing activity (%) was calculated as *N*_top_/*N*_total_ × 100, where *N*_total_ and *N*_top_ represent the number of total flies and the number of flies at the top (over the 5-cm line), respectively. Within the same age, values not sharing the same letter are significantly different (p < 0.05).

To further assess the detrimental effects of tBH on SCA3 phenotypes and to test the protective effects of CA and Res, we studied the survival rates and climbing activity of ELAV-SCA3tr-Q78 flies. Compared with ELAV-SCA3tr-Q27 flies, ELAV-SCA3tr-Q78 flies express an ataxin-3 polyQ tract of 78 residues in neurons, which results in reduced survival rates and climbing activity. These reductions are further worsened by tBH treatment. We found that addition of CA and Res enhanced the survival rates and climbing activity in tBH-treated ELAV-SCA3tr-Q78 flies (Fig. 5).

### CA and Res ameliorate the effects of tBH treatment in ELAV-SCA3tr-Q78 transgenic *Drosophila*

**Figure 6:**
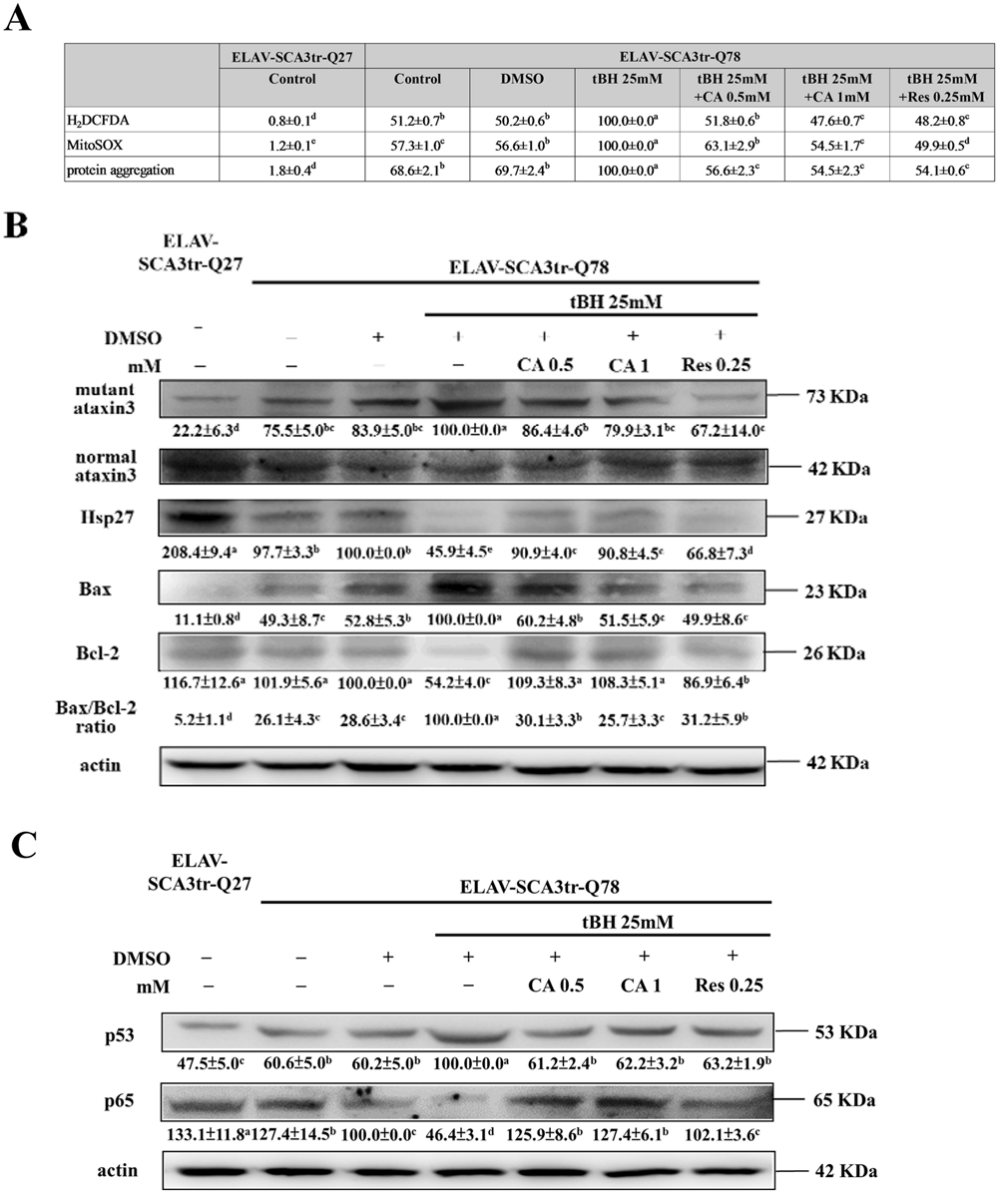
Effects of CA and Res on tBH-treated ELAV-SCA3tr-Q78 transgenic *Drosophila*. **(A)** H_2_DCFDA, MitoSOX, and protein aggregate levels. **(B)** Mutant and normal ataxin-3, Hsp27, Bax, Bcl-2. **(C)** p53 and NF-κB protein expression in 22-day-old male ELAV-SCA3tr-Q78 flies. Values are mean ± SD, n = 50 male flies in three separate experiments. Data are expressed as the percentage of ELAV-SCA3tr-Q78 flies treated with tBH alone. Values not having the same letter are significantly different (p < 0.05).

CA and Res decreased ROS production, mutant ataxin-3 expression, the formation of protein aggregates, and augmented Hsp27 expression in tBH-treated ELAV-SCA3tr-Q78 flies. Moreover, CA and Res restrained the effects of tBH on apoptosis-related protein expression, such as of Bax and Bcl-2, as well as expression of p53 and NF-κB in ELAV-SCA3tr-Q78 flies (Fig. 6).

## Discussion

Evidence suggests that mutant ataxin-3 is associated with an increase in susceptibility to oxidative stress that has a significant impact on neuronal cell death in SCA3^5, 6, 38^. Our data showed that, in response to tBH treatment, a considerable amount of ROS and apoptotic cell death was observed in SK-N-SH-MJD78 cells but not in SK-N-SH or SK-N-SH-MJD26 cells. Most importantly, for the first time, we demonstrated that treatment with CA and Res could attenuate tBH-induced ROS and cell death in SK-N-SH-MJD78 cells and could modulate the molecules related to the mitochondria-mediated apoptotic pathway, such as the expression of Bax and Bcl-2, the Bax/Bcl-2 ratio, mitochondrial transmembrane potential, the cytoplasmic cytochrome *c* level, and activation of the caspase cascade and PARP.

Overexpression of Hsp27 and Bcl-2 can blunt SK-N-SH-MJD78 cells to staurosporine-induced apoptotic stress, likely because Hsp27 and Bcl-2 serve as antioxidant proteins to protect cells against oxidative stress and subsequent apoptosis^33, 34^. Notably, in neuronal and non-neuronal cells, mutant ataxin-3 is causally involved in the down-regulation of Hsp27 and Bcl-2 protein expression by diminishing protein synthesis and mRNA stability, respectively^6, 39^. In the fibroblasts and induced pluripotent stem cells from SCA3 patients, the autophagy is impaired and induction of autophagy by rapamycin decreases mutant ataxin-3 expression^40, 41^. Overexpression of autophagic beclin-1 protein improves autophagosomal flux and clearance of mutant ataxin-3 respectively in rat primary striatal cells and Neuro-2a cells transfected with mutant ataxin-3^36^. Treatment with temsirolimus, an mTOR inhibitor, can improve motor performance in the transgenic mouse of SCA3 through induction of the autophagy pathway leading to diminution of mutant ataxin-3 and its aggregate levels in neurons^35^. In the present work, our data indicated that compared with the expression in SK-N-SH-MJD26 cells, expression of Hsp27 and Bcl-2, and autophagy levels were reduced and expression of mutant ataxin-3 protein and protein aggregates were increased in SK-N-SH-MJD78 cells. Notably, these changes were much more pronounced in SK-N-SH-MJD78 cells treated with tBH. Our data suggest that CA and Res may through upregulation of the autophagy process decrease the expression of mutant ataxin-3 and protein aggregates, resulting in restoration of the Hsp27 and Bcl-2 protein expression in tBH-treated SK-N-SH-MJD78 cells.

A large body of evidence has shown that aberrant activation of the p53 pathway elicits mitochondria-mediated apoptotic neuronal death, which is implicated in the neurodegeneration in several neurological disorders including polyQ diseases^8, 11^. Not only in primary cultures of central nervous cells expressing mutant ataxin-3 but also in pontine nuclei of SCA3 transgenic mice, mutant ataxin-3 can increase the expression of total and phosphorylated p53 as well as the transcriptional activity of p53, which is associated with upregulation of Bax and activated caspase-3 and 9 expression and subsequent apoptotic cell death^9, 10^. Although activation of NF-κB in acute brain injury is neurotoxic, constitutive activation of NF-κB is required for the maintenance of survival of central neurons^14^. Moreover, impairment of NF-κB activity is involved in the neuronal death in SCA7 and Huntington’s disease^17, 18, 42^. Although the exact effect remains unknown, previous data showed that mutant ataxin-3 can interact with IκB-α and NF-κB p65^18^. The results of the present study showed that tBH treatment caused activation of the p53 and inactivation of the NF-κB pathway in SK-N-SH-MJD78 cells and that CA and Res could attenuate the effects of tBH by reverting the transcriptional activities of p53 and NF-κB to control levels. Furthermore, when the ability to recover NF-κB activation was impeded by transfection with a DNM IκB-α plasmid, CA and Res could no longer modulate not only p53 activation but also cell apoptosis and its related molecule expression in tBH-treated SK-N-SH-MJD78 cells. Notably, the preventive effect of CA and Res on mutant ataxin-3, protein aggregates, and Hsp27 levels in tBH-treated SK-N-SH-MJD78 cells was reversed when the cells were transfected with the DNM IκB-α plasmid. These data suggest for the first time that the restoration of NF-κB activity is important in the inhibitory effects of CA and Res on p53 activation and cell death in tBH-treated SK-N-SH-MJD78 cells.

It is well established that MJDtr-Q78 transgenic flies, which exhibit tissue-specific expression of ataxin-3 polyQ tracts of 78 residues, have features similar to human SCA3, with late-onset, progressive neurodegeneration and abnormal protein aggregates. Although due to the neuron-specific promoter elav, no remarkable degeneration of adult eye is observed in ELAV-MJDtr-Q78 flies compared with ELAV-MJDtr-Q27 control flies, the ELAV-MJDtr-Q78 flies have a shorter life span and reduced climbing activity^43, 44^. The data presented here showed that administration of CA and Res prolonged life span and restored locomotor activity during exposure to tBH in ELAV-MJDtr-Q78 flies. Consistent with the *in vitro* findings, CA and Res diminished the effects of tBH on ROS production, protein aggregates, and the protein expression of mutant ataxin-3, Hsp27, and apoptotic-related molecules in ELAV-MJDtr-Q78 flies. Moreover, CA and Res reduced the tBH-induced alterations in p53 and p65 expression in ELAV-MJDtr-Q78 flies.

In addition to size of the polyQ expansion in causative proteins, other genetic or environmental factors are also involved in the variability in age at onset of spinocerebellar ataxia^45^. Selective neuronal cell loss is a common feature of polyQ diseases for which until now no established disease-modifying therapy has been available^46^. The data from the present study demonstrated that CA and Res could amend ROS production, mitochondrial-mediated apoptosis, and p53 and NF-κB activation in both tBH-treated *Drosophila* and human neuroblastoma cell models of SCA3. Notably, we have demonstrated for the first time that CA and Res can restore NF-κB transcriptional activity and that this restoration is associated with decreased neuronal cell death in tBH-treated SK-N-SH-MJD78 cells. Our findings support the beneficial role of CA and Res in diminishing oxidative stress induced neuronal cell death in SCA3. It will be worthy to study the preclinical therapeutic potential of CA and Res in other polyQ diseases.

## Methods

### Materials

The human neuroblastoma cell line SK-N-SH as well as SK-N-SH-MJD26 and SK-N-SH-MJD78 cells stably expressing full-length ataxin-3 with 26 and 78 CAG repeats, respectively, were given by Prof. Mingli Hsieh (Department of Life Science, Tunghai University, Taiwan). The UAS-MJDtr-Q27, UAS-MJDtr-Q78, and *elav-*Gal4 fly strains were obtained from the Bloomington *Drosophila* stock center (Indiana University, Bloomington, IN, USA). CA, Res, and tBH were from Sigma Chemical Company (St Louis, MO). The specific antibodies for hsp27, p53, Bax, Bcl-2, beclin-1, histone H1, glyceraldehyde 3-phosphate dehydrogenase (GAPDH), cytochrome *c*, and total and phosphorylated IKK-α/β and IκB-α were purchased from Santa Cruz Biotechnology (Santa Cruz, CA). Antibodies against total (pro) and cleaved PARP, caspase-9, caspase-7, and phosphorylated p53 were from Cell Signaling Technology Inc. (Beverly, MA). The specific antibodies for p65, caspase-3, LC3 and β-actin were from BD Biosciences (Boston, MA), Novus Biologicals (Littleton, CO), and MBL International (Wobum, MA), EMD Millipore (Billerica, MA), respectively. Antibodies against p62 and ataxin-3 were obtained from Abcam (Cambridge, MA). The Mitochondria/Cytosol Fractionation Kit and Caspase-3 Colorimetric Assay Kit were from BioVision Inc. (San Francisco, CA, USA) and Millipore (Temecula, CA), respectively.

### Cell culture and treatment

Human neuroblastoma SK-N-SH, SK-N-SH-MJD26, and SK-N-SH-MJD78 cells were grown in Dulbecco’s modified Eagle’s medium (DMEM) supplemented with 2 mM glutamine, 1% penicillin/streptomycin, and 1% nonessential amino acids and containing 10% heat-inactivated fetal bovine serum (Invitrogen Corporation, Carlsbad, CA) at 37 °C under a humidified atmosphere of 5% CO_2_. SK-N-SH-MJD26 and SK-N-SH-MJD78 cells were selected in a culture medium supplemented with 0.1 mg/mL G418 (Invivogen, San Diego, CA). When cells reached 90% confluence, the media were replaced with serum-free media with or without 6 µM tBH plus DMSO vehicle control, 3 µM CA, or Res.

### Cell viability assay

The mitochondrial-dependent reduction of 3-(4,5-dimeth-ylthiazol-2-yl)-2,5-diphenyltetrazoliumbromide (MTT) to formazan was used to measure cell respiration as an indicator of cell viability. After 48 h, cells were incubated in DMEM containing 0.5 mg/mL MTT for 3 h. The medium was then removed and isopropanol was added to dissolve the formazan. After centrifugation at 5000 × *g* for 5 min, supernatant from each sample was added to 96-well plates, and the absorbance was read at 570 nm in a VersaMaxTM Tunable Microplate Reader (Molecular Devices Corporation, Sunnyvale, CA).

### Protein extraction and Western blot

Protein extracts from transgenic flies and cells were prepared by using RIPA lysis buffer or the Mitochondria/Cytosol Fractionation Kit. The nuclear proteins were extracted by using hypotonic extraction buffer and then hypertonic extraction buffer^47^. Equal amounts of proteins were denatured and separated on SDS–polyacrylamide gels and were then transferred to polyvinylidene difluoride membranes. The blots were incubated with primary antibodies and horseradish peroxidase-conjugated secondary antibodies. Immunoreactive protein bands were developed by use of an enhanced chemiluminescence kit (Perkin–Elmer Life Science, Boston, MA) and were visualized by use of a luminescent image analyzer (LAS-1000 plus, Fuji Photo Film Company, Japan) and quantified by use of an AlphaImager 2200 gel documentation system (Alpha Innotech Corp., San Leandro, CA).

### Plasmids and transient transfection

The plasmid expressing DNM IκB-α, the pSV-β-galactosidase control vector, and the reporter plasmids of pNF-κB-Luc and pp53-TA-Luc were purchased from Clontech (Palo Alto, CA), Promega Corp. (Madison, WI), and Stratagene Inc. (La Jolla, CA), respectively. The plasmid or control vector was transfected into cells by use of Lipofectamine 2000 transfection reagent (Invitrogen) according to the manufacturer’s instructions.

### Reporter gene assay

NF-κB and p53 transcriptional activity was determined by activity of the reporter enzyme luciferase by using the Luciferase Assay System and was then corrected on the basis of β-galactosidase activity by use of the β-Galactosidase Enzyme Assay System with Reporter Lysis Buffer from Promega Corp.

### Flow cytometry and fluorometry

Cells stained with CM-H_2_DCFDA, TMRE, acridine orange, MitoSOX Red (Invitrogen), and Annexin V: FITC Apoptosis Detection Kit II (BD PharMingen, San Jose, CA) were analyzed with the FACSCalibur flow cytometer (BD Biosciences, Franklin Lakes, NJ). The fluorescence intensity of flies stained with CM-H_2_DCFDA and MitoSOX Red was measured by use of the FlexStation^®^3 Multi-Detection Reader (Applied Biosystems, Foster City, CA). The activity of caspase-3 and protein aggregates was measured by use of Caspase-3 Colorimetric Assay kits (EMD Millipore) and the ProteoStat^®^ protein aggregation assay (Enzo Life Science, Farmingdale, USA), respectively, and the fluorescence intensity was measured by using the FlexStation^®^3 Multi-Detection Reader.

## ProteoStat dye staining

Fluorescent staining of misfolded protein aggregates was performed by using a ProteoStat Aggresome Detection Kit (Enzo Life Science). After fixation with 4% paraformaldehyde, cells were stained with ProteoStat Aggresome Detection Reagent and Hoechst 33342. Cell images were analyzed under a fluorescence microscope (Olympus, Melville, NY, USA).

### *Drosophila* stocks and crosses

UAS-MJDtr-Q27 and UAS-MJDtr-Q78 flies were grown on a standard cornmeal medium at 25 °C on a 12-h light-dark cycle at 60% relative humidity. Virgin female flies carrying the driver *elav-Gal4* on the X chromosome were crossed to male flies carrying UASQ27 or UASQ78, and F1 offspring expressed ataxin-3tr-Q27 or ataxin-3tr-Q78 in the nervous system.

### Survival and climbing

Standard media were added with or without 25 mM tBH plus DMSO vehicle control, 0.5 mM or 1 mM CA, or 0.25 mM Res and were changed every 3 days. Survival rates and climbing activity were measured every 3 days. The number of dead flies was counted and survival rates were graphed and compared for statistical differences by using Kaplan-Meier log-rank analysis among test groups (SigmaStat V3.5 software; Systat Software, Inc., San Jose, CA). For measurement of climbing activity, flies were tapped down and the number of flies that climbed up 5 cm in 18 s was recorded. The climbing activity (%) was calculated as *N*_top_/*N*_total_ × 100, where *N*_total_ and *N*_top_ represented the numbers of total flies and flies at the top (over the 5-cm line), respectively.

### Statistical analysis

Data are expressed as the means ± SDs from at least three independent experiments. Differences among treatments were analyzed by one-way ANOVA and Tukey’s multiple-range test by using the Statistical Analysis System (Cary, NC, USA). A value of p < 0.05 was considered to be statistically significant.
